# Is workplace-based assessment achievable in practice?

**DOI:** 10.1177/10398562231167686

**Published:** 2023-03-27

**Authors:** Alex Holmes

**Affiliations:** Melbourne, VIC

Dear Sir,

The cessation of independent clinical assessment by the RANZCP is a critical decision for a specialist college. British and Canadian Colleges and the American board still require independent clinical assessments. This change occurred in response to stakeholder forums and as a commitment to contemporary approaches to assessment in medical education.

Competency-Based Education (CBE) has become the predominant paradigm in medical education. It delivers staged programs which emphasise supervised, practice-based learning, enhanced by feedback. Workplace-based assessment (WBA) is integral to CBE. It shifts focus from what the trainee knows to what they do. Formative assessment is devised to aid learning whilst summative assessment aims to measure what has been learned.^1^

The RANZCP summative assessment has evolved representing improvements in fairness, uniformity, reliability and breadth. Observed Structured Clinical Exams replaced Observed Clinical Interviews which superseded long cases. The movement to WBA reflects the natural evolution summative assessment within a CBE framework. In addition to the ideological consistency, WBA has the advantages of practicality, flexibility, efficiency and multi-modality ('holism'). It also alleviates the burden of conducting independent exams and addresses evidence that high stakes summative assessment can impair learning and assessment outcomes.^2^

The disadvantages of WBA relate to training and supervisor burden. Evidence indicates that supervisors and trainees often are inadequately trained in assessment, do not understand the purpose, and interact in settings with insufficient time.^3^ This is particularly challenging in a stretched public health system. Further, the admixture of assessment and supervision is particularly problematic. The capacity for containment, personal reflection and care are not easily maintained when there is a concurrent requirement to judge competence.

Currently, in practice, the predominant solution to an underperforming trainee is that they are determined to be 'good enough' to progress, albeit with room for improvement. That improvement, however, is not guaranteed. Some psychiatrists have already observed decreased ability to assess, synthesise and present cases in a timely and organised manner following the cessation of the independent OCI. (Notably, there is no evidence either way.)

The educational argument for workplace summative assessment is far from conclusive. Without independent assessment, it is likely that the quality and value of core clinical skills will become diminished. As an independent guild we have an obligation to maintain standards. To not do so risks our leadership role in mental health care and our identity as medical specialists.
